# Linear and Nonlinear Optical Properties of an AlSb/InAs/AlSb Spherical Quantum Dot in a Magnetic Field

**DOI:** 10.3390/nano16181152

**Published:** 2026-09-14

**Authors:** Sake Wang, Hassen Dakhlaoui, Bryan M. Wong

**Affiliations:** 1 College of Science, Jinling Institute of Technology, 99 Hongjing Avenue, Nanjing 211169, China; 2 Physics Department, Nanomaterials Technology Unit, Basic and Applied Scientific Research Center (BASRC), College of Science of Dammam, Imam Abdulrahman Bin Faisal University, Dammam 31441, Saudi Arabia; 3 Department of Chemistry, Department of Physics & Astronomy, and Materials Science & Engineering Program, University of California-Riverside, Riverside, CA 92521, USA

**Keywords:** quantum dot, donor impurity, magnetic field, Schrödinger equation, optical absorption coefficients, refractive index changes

## Abstract

We investigate the optical and electronic properties of a spherical AlSb/InAs/AlSb core/shell/shell quantum dot (QD) under the combined effects of size confinement and an external magnetic field. The system contains a centrally located hydrogenic donor impurity whose attractive Coulomb interaction affects the distribution of electronic energy levels, their corresponding wavefunctions, and their optical responses. The total optical absorption coefficients and refractive index changes are analyzed as functions of incident photon energy by systematically varying the core radius and the applied magnetic field. The results reveal a tunable optical absorption resonance associated with the $E_{1}\to E_{2}$ intersubband transition, whose spectral position and amplitude are strongly modified by the core radius, magnetic-field strength, and presence of the hydrogenic impurity. Increasing the magnetic field intensity from 0 to 30 T redshifts the optical absorption and refractive index peaks by 20–60 meV, reflecting the predominance of magnetic effects in the total confining potential. In contrast, expanding the internal quantum dot radius (core radius) also leads to a redshift, but in a lower-energy range of incident photons (0–20 meV), governed primarily by the quantum-size confinement effect. This net distinction in energy-shift ranges indicates that applying a magnetic field offers a broader tuning mechanism than quantum-size effects in the proposed core/shell/shell QDs. Furthermore, the coexistence of two distinct absorption ranges underscores the potential of AlSb/InAs/AlSb spherical QDs for various technological applications, including multi-band, magnetically tunable infrared and THz-infrared optical photodetectors.

## 1. Introduction

Low-dimensional quantum heterostructures (LDQHs) continue to attract significant attention due to their tunable optoelectronic properties [1,2,3,4,5,6,7,8,9,10,11,12,13,14,15]. Amongst these heterostructures, quantum wells exhibit electrons that are free to move in one dimension [4,5], quantum wires enable confinement in two dimensions [6,7,8,9,10,11], and quantum dots (QDs) are characterized by three-dimensional confinement [12,13,14,15]. These features can be adjusted either by altering the quantum size and geometric form of the confining potential or by applying external electric/magnetic fields or intense lasers. Furthermore, the tunability of hydrostatic pressure and temperature, as well as the inclusion of planar doping or hydrogenic impurities, can enable control over the aforementioned properties [16,17,18,19,20,21,22,23,24].

LDQHs have been synthesized through well-controlled fabrication methods, including sol–gel techniques, molecular beam epitaxy (MBE), and hydrothermal methods, enabling researchers to obtain structures with dimensions in the range of 10–15 nm [25,26,27,28,29,30] and various geometries [31,32,33,34,35]. QDs are primarily fabricated from semiconductor compounds composed of atoms from the II–VI, III–V, and IV–VI groups. However, in recent years, extensive attention has focused on ZnS/CdS core–shell QDs due to their broad and intensive applications in optoelectronics, photovoltaics, and biomedical domains [36,37,38,39]. For instance, Linkov et al. showed that multi-core/shell CdSe/ZnS/CdS/ZnS spherical QDs can be used as fluorescent probes to design and develop biosensors for cancer detection and diagnosis, thereby enabling earlier treatment with anticancer amalgams based on acridine derivatives [40]. Zeiri et al. demonstrated that the linear and nonlinear optical susceptibilities of CdS/ZnS/CdS/ZnS core/shell/well/shell spherical QDs can be modulated by varying the shell size [41]. Finally, Kuzyk et al. showed that QDs used in CdSe/ZnS/CdS/ZnS–albumin complexes produced a substantial lattice distortion, leading to an estimated 40 meV shift in the conduction-band edge [42]. On this basis, the authors proposed potential applications in biosensing technologies aimed at quantifying albumin levels.

Despite numerous theoretical and experimental studies on the electronic properties of core/shell CdSe/ZnS and CdS/ZnS QDs, other semiconductor materials remain underexplored. In particular, exploring new types of core/shell/shell QDs based on AlSb/InAs materials could open a new path for quantum engineering beyond conventional II-IV materials, with potential implications for quantum information technologies, infrared optoelectronics, and strain-sensitive nanodevices.

The present paper aims to address this gap by studying a core/shell/shell AlSb/InAs spherical QD with a hydrogenic impurity and subjected to external magnetic fields. Although the electronic and optical properties of ZnS/CdS/ZnS core/shell/shell spherical QDs have been investigated previously [43], the present investigation focuses on InAs/AlSb materials, which operate in the low-energy regime for designing optoelectronic devices in the infrared/terahertz range. Specifically, InAs has a smaller electron effective mass and promising infrared optical properties, and AlSb forms a higher barrier potential that guarantees strong electron confinement within the InAs region. Due to quantum confinement between the semiconductor layers, the carriers’ energy levels in the QD can be widely tuned by structural parameters such as the core/shell quantum size, the application of external electromagnetic fields, and the conduction (valence) band discontinuities between the layers. These physical factors facilitate precise tunability of electronic properties and optical responses, making these structures useful in modern applications spanning optoelectronics, medicine, chemistry, and renewable energy.

## 2. Theory

Within the effective-mass framework, the Hamiltonian describing the total energy of a confined electron in a spherical AlSb/InAs/AlSb core/shell/shell QD without the presence of a magnetic field and impurities can be written as [43]:

(1) $$H^{0}=\frac{{\vec{p}}^{2}}{2m*}+V_{conf}\left(r\right).$$

where $\vec{p}$ is the electron momentum, m* denotes the effective mass of the electron, and $V_{conf}(r)$ represents the spherically confining potential. Both m*(r) and $V_{conf}(r)$ vary with the radial coordinate of the QD and can be written as follows [44]:

(2) $$m*\left(r\right)=\left\{\begin{array}{c} m*\left(AlSb\right),\;\mathrm{if}\;0\leq r\leq R_{1}\\ m*\left(InAs\right),\;\mathrm{if}\;{\;R}_{1}<r\leq R_{2}\\ m*\left(AlSb\right),\;\mathrm{if}\;R_{2}<r\leq R_{3}\; \end{array}\right.$$

and

(3) $$V_{conf}\left(r\right)=\left\{\begin{array}{c} V_{0},\;\mathrm{if}\;0\leq r\leq R_{1}\\ 0,\;\mathrm{if}\;{\;R}_{1}<r\leq R_{2}\\ V_{0},\;\mathrm{if}\;R_{2}<r\leq R_{3}\;\\ \infty ,\;\mathrm{if}\;r>R_{3}\; \end{array}\right.$$

The total Hamiltonian describing the electron motion within the core/shell/shell QD, including a hydrogenic impurity at its center and subjected to an external magnetic field, can be written as follows [43]:

(4) $$H=\frac{1}{2m*}{\left(\vec{p}+\;e\vec{A}\right)}^{2}+V_{conf}\left(r\right)-\frac{Ze^{2}}{\varepsilon \left|\vec{r}\right|},$$

where $\vec{A}$, e, and ε are the magnetic vector potential, elementary charge, and dielectric constant, respectively. The last term in Equation (4) is the electrostatic attraction between the hydrogenic impurity and the free electron. In the absence of impurity, Z=0, while its presence is considered by setting Z equal to 1. We define the vector potential $\vec{A}$ so that the magnetic field is given by $\vec{B}=\vec{\nabla }\;\times \;\vec{A}$. Under the Coulomb gauge condition $\left(\vec{\nabla }\cdot \vec{A}=0\right)$, the potential vector is obtained from the relation: $\vec{A}=\frac{1}{2}(\vec{B}\times \vec{r})$. Assuming the magnetic field to be oriented along the z-direction $\left(\vec{B}=B{\vec{u}}_{z}\right)$, and invoking azimuthal symmetry, the solution of the previous equation can be obtained in cylindrical coordinates by adopting the wave function in the form $\psi \left(\rho ,\phi ,z\right)=R(\rho ,z)e^{im\phi }$. Subsequently, the energy levels of the electron for the system without $\left(Z=0\right)$ and with $\left(Z=1\right)$ an impurity, along with its corresponding wavefunctions, are computed numerically by solving the 2D Schrödinger equation [43]:

(5) $$\left[-\frac{\hbar^{2}}{2m*\left(r\right)}\nabla_{\rho ,z}^{2}\;+\;\frac{m^{2}\hbar^{2}}{2m*\left(r\right)\rho^{2}}\;+\;\frac{\hbar eBm}{2m*\left(r\right)}\;+\;\frac{e^{2}B^{2}\rho^{2}}{8m*\left(r\right)}\;+\;V_{conf}\left(r\right)\;-\;\frac{Ze^{2}}{\varepsilon \sqrt{\rho^{2}+z^{2}}}\right]\;\times \;R\left(\rho ,z\right)\;=\;E_{m}R\left(\rho ,z\right),$$

where $\nabla_{\rho ,z}^{2}$ is the two-dimensional Laplacian operator in cylindrical coordinates, $r=\sqrt{\rho^{2}+z^{2}}$ is the electron–impurity distance, and m is the angular quantum number. When both a magnetic field and a hydrogenic impurity are considered, the analytical solution of Equation (5) is not possible, and a numerical solution is obtained with the finite difference method. This procedure is carried out on a uniform rectangular grid spanning the computational domain defined as: $0\leq \rho \leq R_{3}$ and $-R_{3}\leq z\leq R_{3}$; with spacing $h=R_{3}/(M+1)$ and $k={2R}_{3}/(N+1)$, respectively, where M and N are taken as large integers. Therefore, the mesh is defined as follows: $\left(\rho_{i},z_{j}\right)=(\left(i-1\right)h,\left(j-1\right)k)$, with 1≤i≤M+2 and 1≤j≤N+2.

Due to the inherent spherical symmetry of the system, the first-order optical absorption coefficient $\alpha^{1}(\omega )$ can be written as follows [43]:

(6) $$\alpha^{1}\left(\omega \right)=\omega \sqrt{\frac{\mu }{\varepsilon_{r}}}\frac{{\left|M_{12}\right|}^{2}\sigma \;\hbar T_{12}}{{\left(E_{2}-E_{1}-\hbar \omega \right)}^{2}+{\left(\hbar T_{12}\right)}^{2}}.$$

The third-order absorption coefficient can be obtained from the following formula:

(7) $$\alpha^{\left(3\right)}\left(\omega ,I\right)=-2\omega \sqrt{\frac{\mu }{\varepsilon_{r}}}\left(\frac{I}{\varepsilon_{0}n_{r}c}\right)\left[\frac{{\left|M_{12}\right|}^{4}\sigma \;\hbar T_{12}}{{\left[{\left(E_{12}-\hbar \omega \right)}^{2}+{\left(\hbar T_{12}\right)}^{2}\right]}^{2}}\right.\;-\left.\frac{{\left|M_{22}{-M}_{11}\right|}^{2}}{{\left|{2M}_{12}\right|}^{2}}\frac{{\left|M_{12}\right|}^{4}\sigma \;\hbar T_{12}}{{\left[{\left(E_{12}-\hbar \omega \right)}^{2}+{\left(\hbar T_{12}\right)}^{2}\right]}^{2}}\frac{\left(3E_{12}^{2}-4E_{12}\hbar \omega +\hbar^{2}\left(\omega^{2}-{T_{12}}^{2}\right)\right)}{E_{12}^{2}+{\left(\hbar T_{12}\right)}^{2}}\right].$$

The net absorption coefficient is given by the combination of the linear term and the third-order nonlinear term according to the following expressions [43]:

(8) $$\alpha \left(\omega ,I\right)=\alpha^{\left(1\right)}\left(\omega \right)+\alpha^{\left(3\right)}\left(\omega ,I\right).$$

The corresponding formulas for computing the relative refractive index changes are given by [43]:

(9) $$\frac{{∆n}^{\left(1\right)}\left(\omega \right)}{n_{r}}=\frac{\sigma {\left|M_{12}\right|}^{2}}{2n_{r}^{2}\varepsilon_{0}}\times \left[\frac{E_{12}-\hbar \omega }{{\left(E_{12}-\hbar \omega \right)}^{2}+{\left(\hbar T_{12}\right)}^{2}}\right],$$

(10) $$\frac{∆n^{\left(3\right)}\left(\omega ,I\right)}{n_{r}}=\frac{-\mu c}{4n_{r}^{3}\varepsilon_{0}}\frac{{\left|M_{12}\right|}^{2}\sigma \;I}{{\left[{\left(E_{12}-\hbar \omega \right)}^{2}+{\left(\hbar T_{12}\right)}^{2}\right]}^{2}}\times \left[4\left(E_{12}-\hbar \omega \right){\left|M_{12}\right|}^{2}-\frac{{\left(M_{22}-M_{11}\right)}^{2}}{E_{12}^{2}+{\left(\hbar T_{12}\right)}^{2}}\times \left[\left(E_{12}-\;\hbar \omega \right)\left(E_{12}\left(E_{12}-\hbar \omega \right)-{\left(\hbar T_{12}\right)}^{2}\right)-{\left(\hbar T_{12}\right)}^{2}\left(2E_{12}-\hbar \omega \right)\right]\right],$$

(11) $$\frac{∆n\left(\omega ,I\right)}{n_{r}}=\frac{∆n^{\left(1\right)}\left(\omega \right)}{n_{r}}+\frac{∆n^{\left(3\right)}\left(\omega ,I\right)}{n_{r}}.$$

The previous expressions are derived using the density matrix approach, as described in Ref. [45]. $E_{12}=E_{2}-E_{1}$ denotes the energy separation between the ground “1” and first-excited “2” states. Moreover, $M_{12}$ is the reduced off-diagonal dipole matrix element involving the two lowest wavefunctions; for incident light polarized along the z-axis, it is given by the expression [43]:

(12) $$M_{12}=\langle \psi_{1}\left(r,\phi ,z\right)|z|\psi_{2}\left(r,\phi ,z\right)\rangle .$$

The $\hbar T_{12}$ term is the damping factor associated with the finite electron lifetime produced by the intersubband dispersion, which we set to 0.7 meV throughout this study. In addition, the term $n_{r}$ indicates the relative refractive index, $\mu_{0}$ is the permeability of the free space, and σ is the carrier density set at $3\times 10^{22}$ $m^{-3}$. Finally, I denotes the intensity of the optical radiation applied to the system, which we set to 40 MW/$m^{2}$.

The ground state binding energy, which describes the electrostatic interaction energy between the electron and the on-center hydrogenic impurity, is evaluated using the following relation [43]:

(13) $$E_{b}=E_{z=0}-E_{z=1},$$

where $E_{z=0}$ and $E_{z=1}$ denote the ground-state energies of the QD system in the absence (z=0) and presence (z=1) of impurity, respectively. The other parameters employed in this study are [44]: $m*\left(InAs\right)=0.023m_{0};\;m*\left(AlSb\right)=0.14m_{0};\;V_{0}=1.35\;eV;\;\mu_{0}=4\pi \times 10^{-7}$ $TmA^{-1}$.

The magnetic field range [0–30 T] was chosen to study the regime where magnetic confinement appreciably modifies the electronic and optical properties. Analogous or upper magnetic fields have been considered in previous theoretical studies of related semiconductor nanostructures [46,47], while high-field magneto-optical experiments demonstrate their availability at specific facilities [48,49,50].

## 3. Results and Discussions

**Figure 1:**
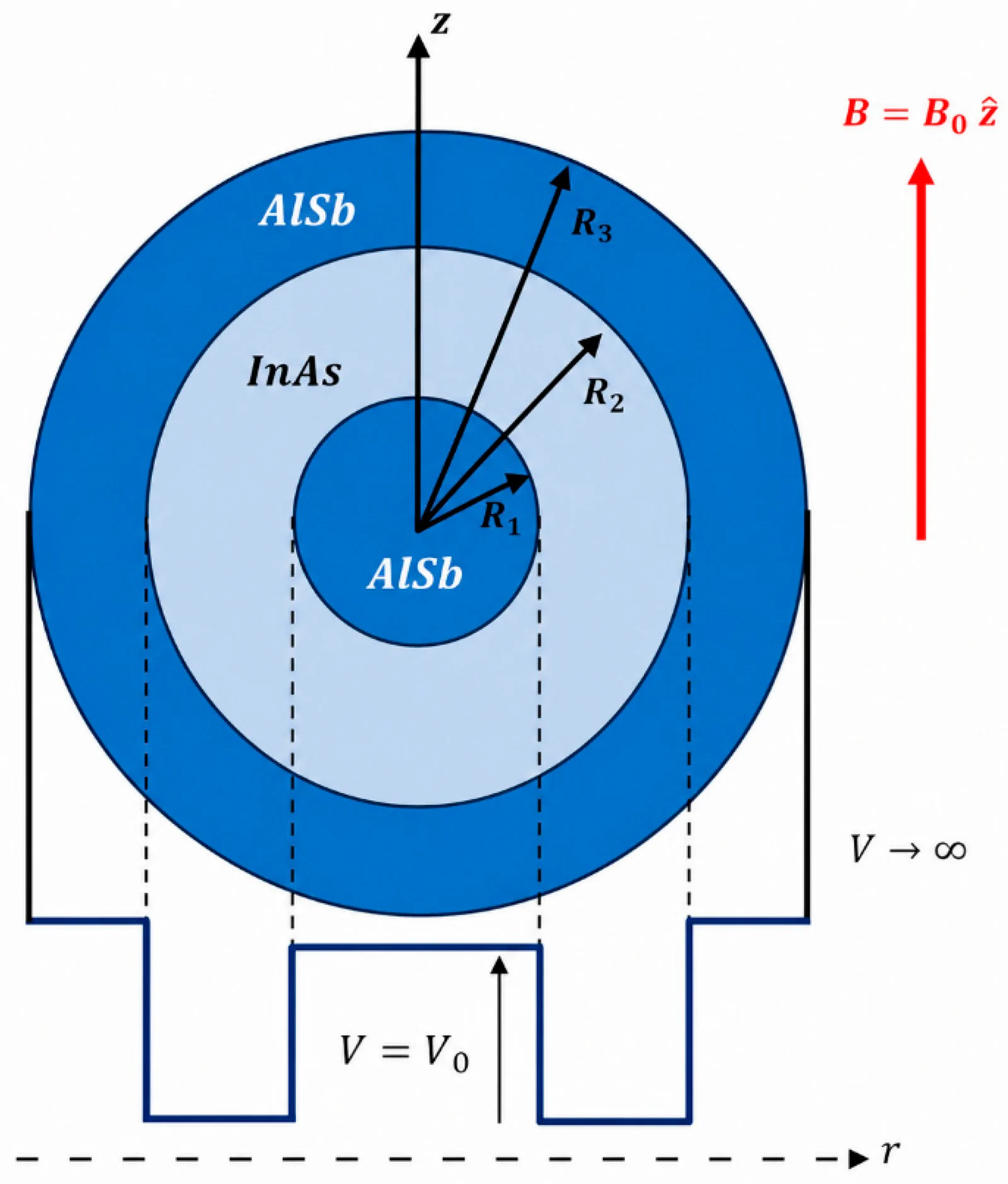
Schematic diagram of the AlSb/InAs/AlSb spherical QD.

**Figure 2:**
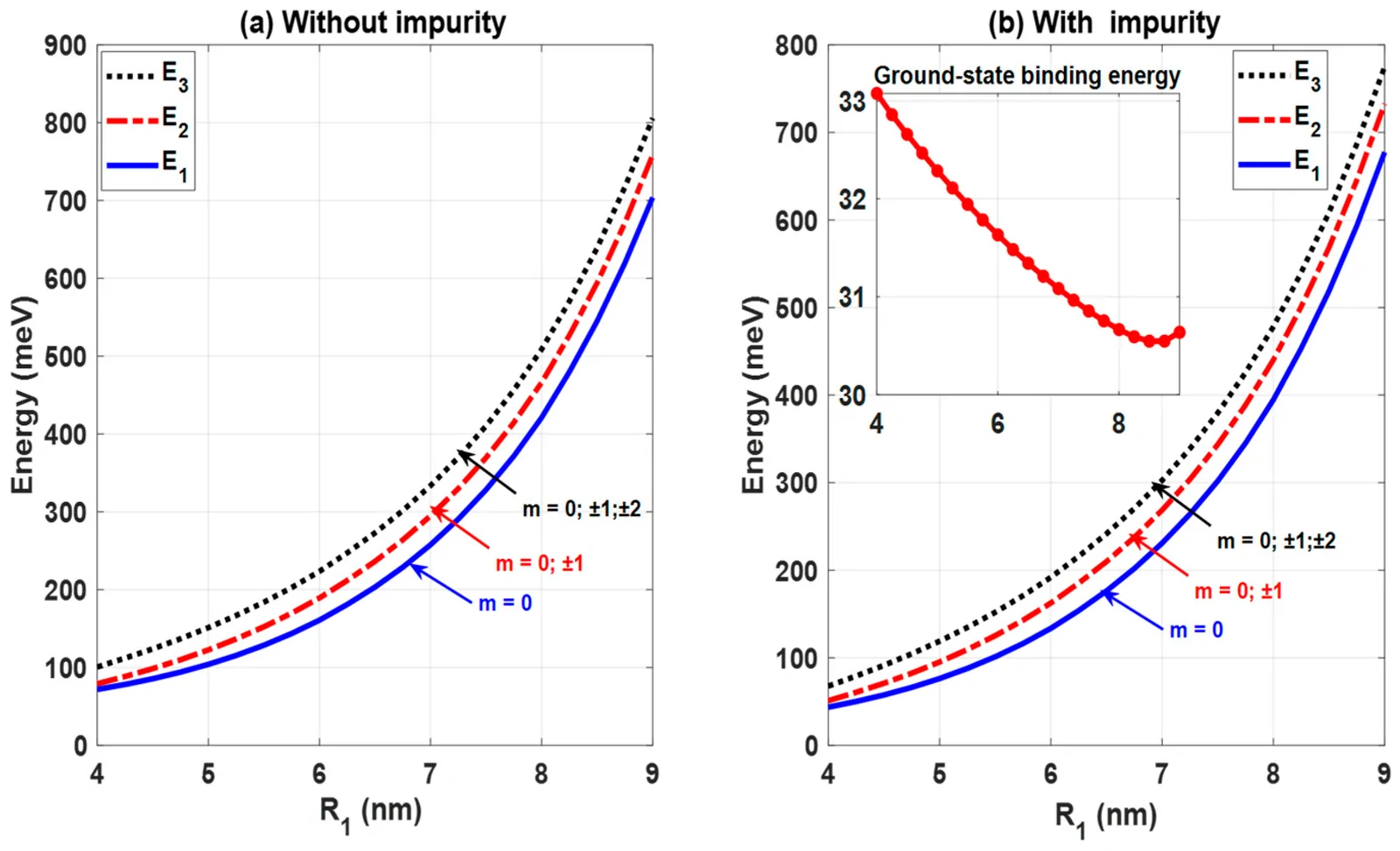
Lowest three electronic energy levels in the AlSb/InAs/AlSb core/shell/shell spherical QD as a function of core radius $R_{1}$ (**a**) without impurity, and (**b**) with impurity for $R_{2}=13$ and $R_{3}=14$ nm. The inset figure in (**b**) represents the binding energy of the ground state.

Figure 1 shows the system under investigation, which consists of a spherical AlSb/InAs/AlSb core/shell/shell QD embedded in an infinite potential well and a hydrogenic impurity at its center. The radii of the core, intermediate shell, and outer shell are $R_{1}$, $R_{2}$, and $R_{3}$, respectively. The bottom of Figure 1 displays a schematic representation of the confining potential as a function of the radial coordinate r. This potential is set to zero in the InAs region, $V_{0}$ in the AlSb material, and infinity in the vacuum surrounding the entire system. Figure 2 illustrates the dependence of the lowest three energy levels on the core radius $R_{1}$ for the AlSb/InAs/AlSb core/shell/shell QD without a magnetic field (B=0). The variations are given for both configurations: without a donor impurity (Figure 2a) and with a donor impurity located at the center of the QD (Figure 2b). Both figures show that these lowest energy levels increase with $R_{1}$, which can be attributed to the reinforcement of quantum confinement. As the core radius $R_{1}$ increases, the thickness of the InAs shell, where the electron is mostly localized, decreases. Consequently, the electron is confined to a limited spatial region, thereby increasing its kinetic energy and shifting the energy levels upward. This behavior is consistent with the qualitative implication of the Heisenberg uncertainty principle, according to which stronger spatial localization is accompanied by an increase in momentum uncertainty and, hence, in kinetic energy. Figure 2b illustrates the impact of the hydrogenic impurity positioned at r=0. The presence of impurities attracts free electrons and shifts their energy levels to lower values due to electrostatic interactions. The electron–impurity separation reaches its minimum in the ground state, which accounts for the larger downward energy shift observed for this state.

As shown in the inset of Figure 2b, the ground-state binding energy depends on the internal radius $R_{1}$ and decreases as this radius increases. For instance, at $R_{1}=4$ nm, the binding energy is 33 meV, whereas at $R_{1}=8$ nm, it is only 30.6 meV. This occurs because a larger radius $R_{1}$ pushes the free electron away from the impurity, reducing the electrostatic attraction.

**Figure 3:**
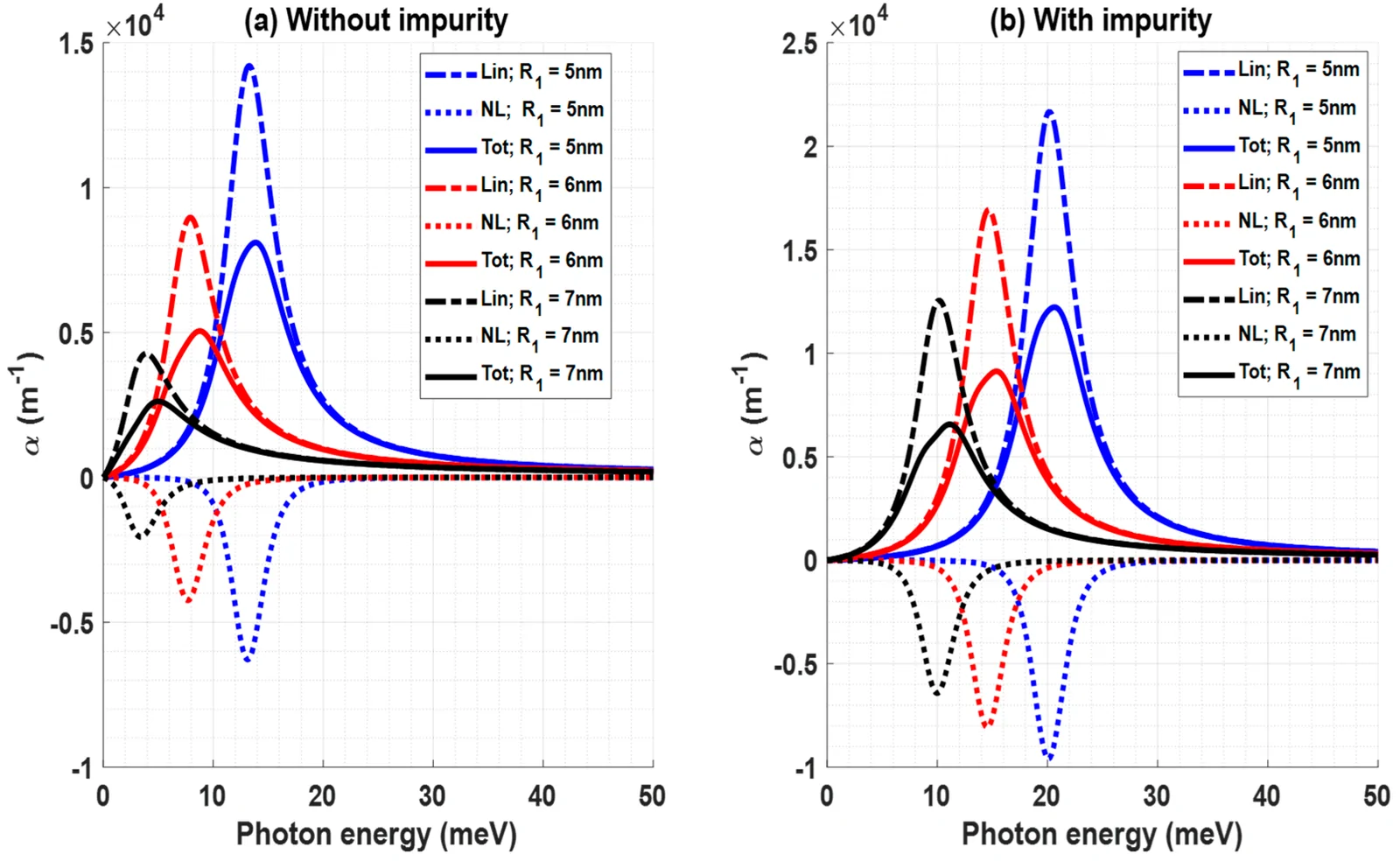
Total (solid line), linear (dashed line), and nonlinear (dotted line) optical absorption coefficient as a function of incident energy for three values of $R_{1}$ (**a**) without the impurity, and (**b**) with the impurity for $R_{2}=13$ and $R_{3}=14$ nm.

**Figure 4:**
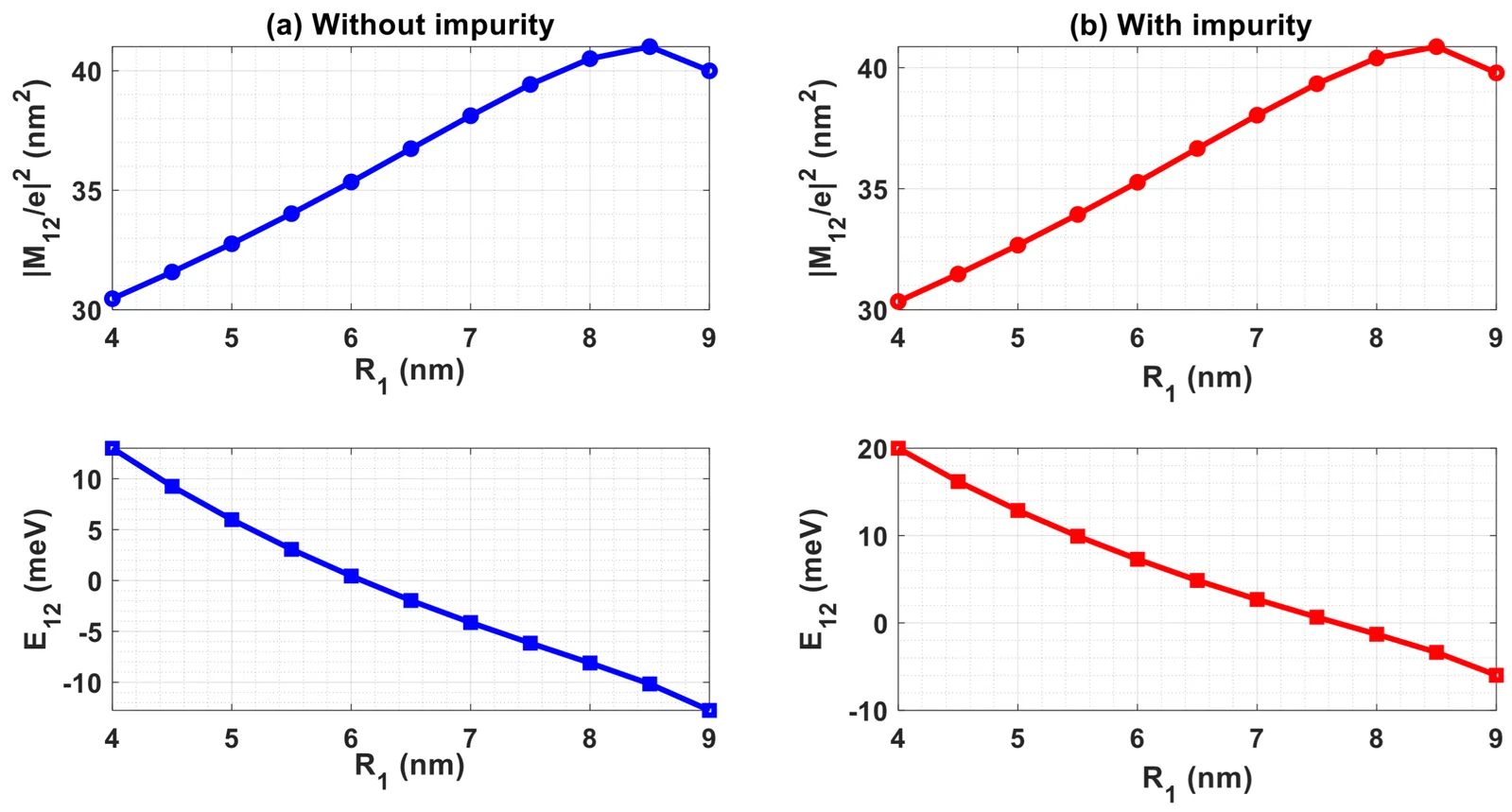
Variation of ${\left|M_{12}\right|}^{2}$ and $\left(E_{12}=E_{2}-E_{1}\right)$ as a function of the core radius $R_{1}$ (**a**) without impurity, (**b**) with impurity. $R_{2}=13$ and $R_{3}=14$ nm.

Because of the spherical symmetry of the confining potential and the magnetic field applied along the z-axis, the energy levels in both figures display the same degeneracies regardless of the presence or absence of the hydrogenic impurity. For instance, the first-excited state is triply degenerate (m=0, ±1), and the second-excited state exhibits five-fold degeneracy (m=0, ±1, ±2). Since we focus on the first transition between the ground and the first excited state for m=0, Figure 3a,b plot the linear, nonlinear, and total optical absorption coefficients as a function of the incident photon energy, both with and without the presence of the hydrogenic impurity for three internal radii $R_{1}=5$, 6, and 7 nm. We observe a decrease in the amplitude for the linear coefficient $\alpha^{1}\left(\omega \right)$ with increasing $R_{1}.$ From Equation (7), the $\alpha^{1}\left(\omega \right)$ maximum is completely dominated by the variation of $E_{12}$ and ${\left|M_{12}\right|}^{2}$, which display opposite variations, as shown in Figure 4a,b. $E_{12}$ decreases as $R_{1}$ increases; in addition, ${\left|M_{12}\right|}^{2}$ increases because of the higher overlap between the two wavefunctions, which are strongly confined in the same region ($R_{1}\leq r\leq R_{2})$. Note that $E_{12}$ decreases more rapidly than ${\left|M_{12}\right|}^{2}\;$ increases, leading to a reduction in the $\alpha^{1}\left(\omega \right)$ amplitude. Furthermore, its redshift is only related to the decrease in the energy separation, $E_{12}$.

In contrast to the linear absorption, the third-order coefficient $\alpha^{3}\left(\omega \right)$ increases with $R_{1}$. This trend is explained by Equation (8), which shows that $\alpha^{3}\left(\omega \right)$ is governed principally by $E_{12}$ and mostly ${\left|M_{12}\right|}^{4}$ due to its fourth-power dependence. As such, the total coefficient $\alpha \left(\omega ,I\right)$ demonstrates a lowering in its amplitude and a red shift as the inner radius increases.

**Figure 5:**
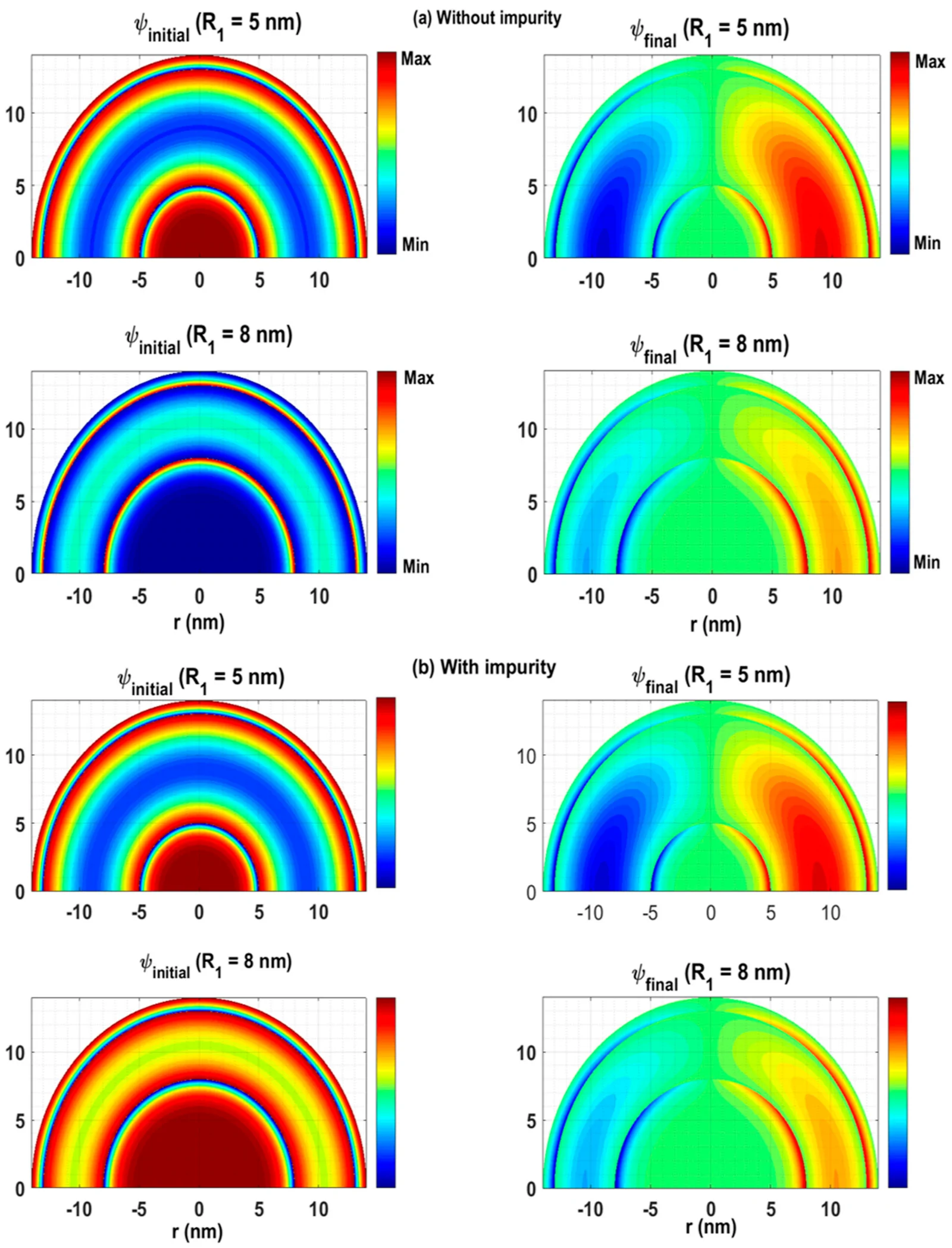
Contour map plots of the initial and final wavefunctions (**a**) without the impurity, and (**b**) with the impurity for $R_{2}=13$ and $R_{3}=14$ nm.

To elucidate the effects of the hydrogenic impurity, Figure 5 plots the two lowest wavefunctions as a function of $\left(r,\phi \right)$ for two different values of $R_{1}$, with and without impurity. By increasing $R_{1}$, the confinement InAs region is reduced, which in turn affects the spread of the two wavefunctions and modifies their spatial distributions. This behavior is consistent with that observed in Figure 2a, where increasing $R_{1}$ from 4 to 9 nm moves the lowest three energy levels to higher values. For a system without the impurity (Figure 5a), the geometrical spread of the two wavefunctions is governed principally by the quantum size of the layers and the confining potential value in each layer. As shown in the right column, the final wavefunction, $\psi_{final}(R_{1}=5\;nm)$, displays a more complex spatial spreading than $\psi_{initial}(R_{1}=5\;nm)$, with minimum and maximum parts characterizing the antisymmetric behavior of an excited state. Figure 5b depicts the spread of the initial and final wavefunctions when the hydrogenic impurity is added at the center of the system. The attractive electrostatic force between the electron and the impurity pushes the electron cloud toward the center of the QD, causing the free electrons to be more localized around the impurity location, (r=0 nm). Figure 5b shows that the ground-state wavefunction is more attracted by the impurity than the excited one, due to its reduced average electron–impurity distance.

**Figure 6:**
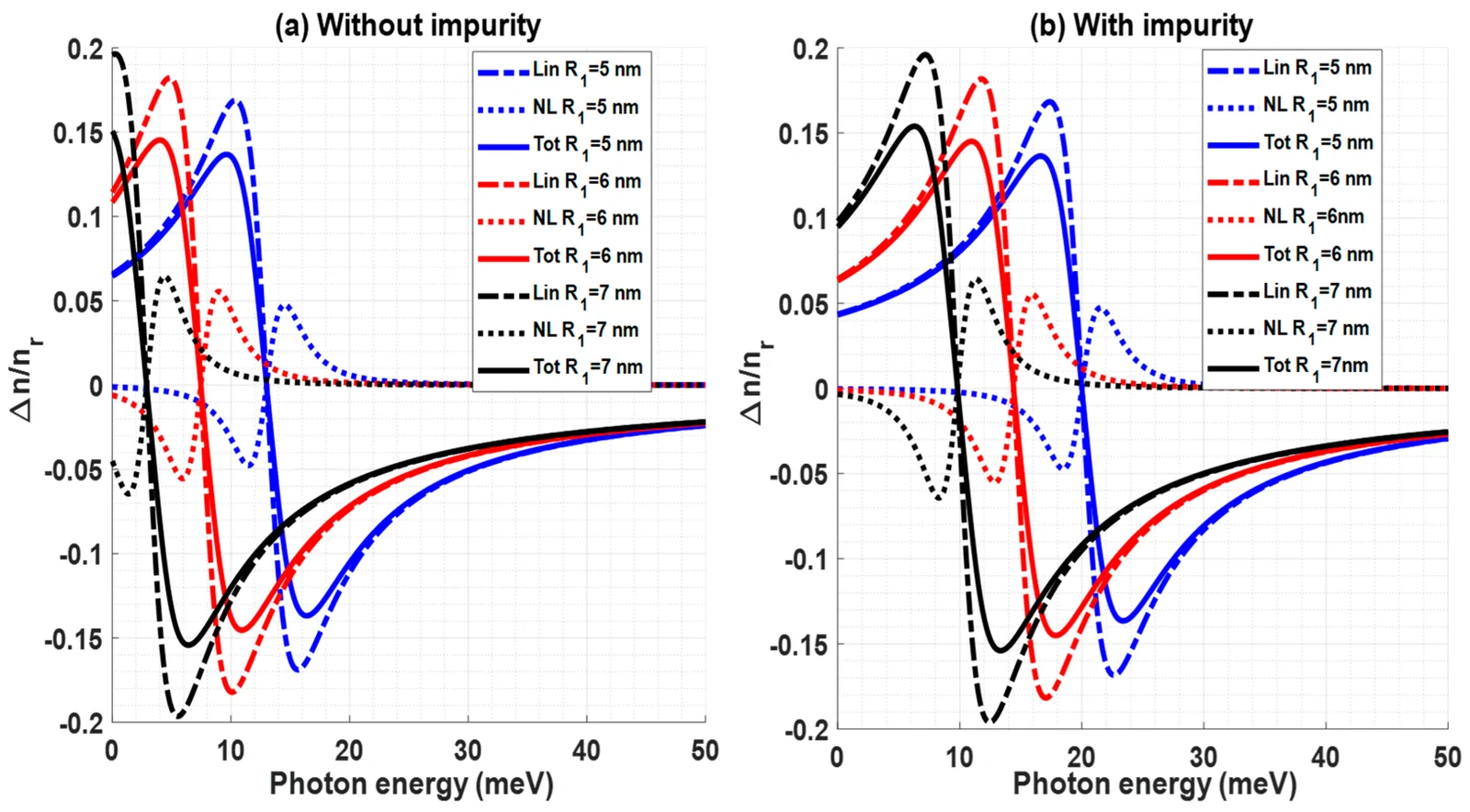
Total (solid line), linear (dashed line), and nonlinear (dotted line) refractive index changes as a function of incident energy for three values of $R_{1}$ (**a**) without the impurity, and (**b**) with the impurity for $R_{2}=13$ and $R_{3}=14$ nm.

Figure 6a,b show the linear $∆n^{\left(1\right)}\left(\omega \right)/n_{r}$, third-order nonlinear $∆n^{\left(3\right)}\left(w\right)/n_{r}$, and the total refractive index changes $∆n\left(\omega \right)/n_{r}$ as a function of incident photon energy for three internal radii. All contributions show a displacement toward lower energies (red shift) when $R_{1}$ increases from 5 to 7 nm. Furthermore, the amplitudes of both contributions (linear and nonlinear) increase with $R_{1}$ in the presence or absence of impurity. This trend is caused by the strengthening of the dipole matrix element and the reduction in the energy separation $E_{12}$. Note that the nonlinear contribution displays an opposite sign to the linear one, which results in a reduction in the total absorption coefficient for all radii.

**Figure 7:**
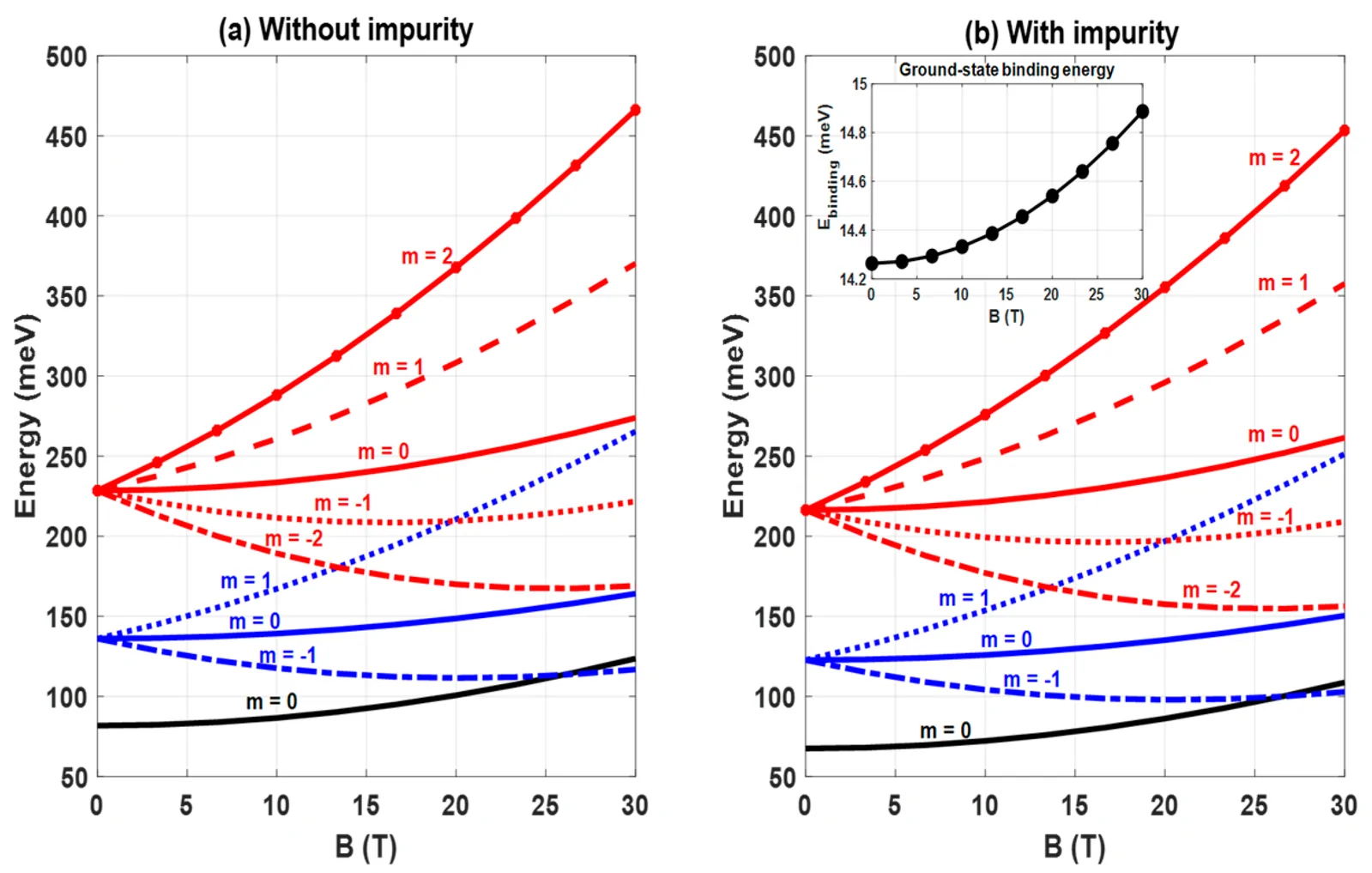
Lowest three electronic energy levels in the AlSb/InAs/AlSb core/shell/shell spherical QD as a function of the applied magnetic field (**a**) without the impurity, and (**b**) with the impurity for $R_{1}=5$, $R_{2}=10$, and $R_{3}=15$ nm. The inset figure in (**b**) represents the binding energy of the ground state (m=0).

Figure 7a,b illustrate the variation in the lowest three energy levels in the AlSb/InAs/AlSb core/shell/shell spherical QD as a function of the applied magnetic field, both in the presence and absence of the on-center hydrogenic impurity for various magnetic numbers m. These figures show that the triple degeneracy in the first excited state and the five-fold degeneracy of the second excited state are removed by increasing the magnetic field between 0 and 30 T. Furthermore, by examining the evolution of energy levels corresponding to positive and negative magnetic numbers m, the energy levels with positive m increase with the applied magnetic field. In contrast, for negative m-states, the energy levels initially decrease with the magnetic field, reaching a certain minimum, and subsequently begin to increase. Therefore, the energy spectrum exhibits an alteration caused by the increase in the magnetic field, where the ground state loses its *s*-like symmetry. Note that at zero magnetic field, the first and the second excited states exhibit three and five order degeneracies, respectively.

**Figure 8:**
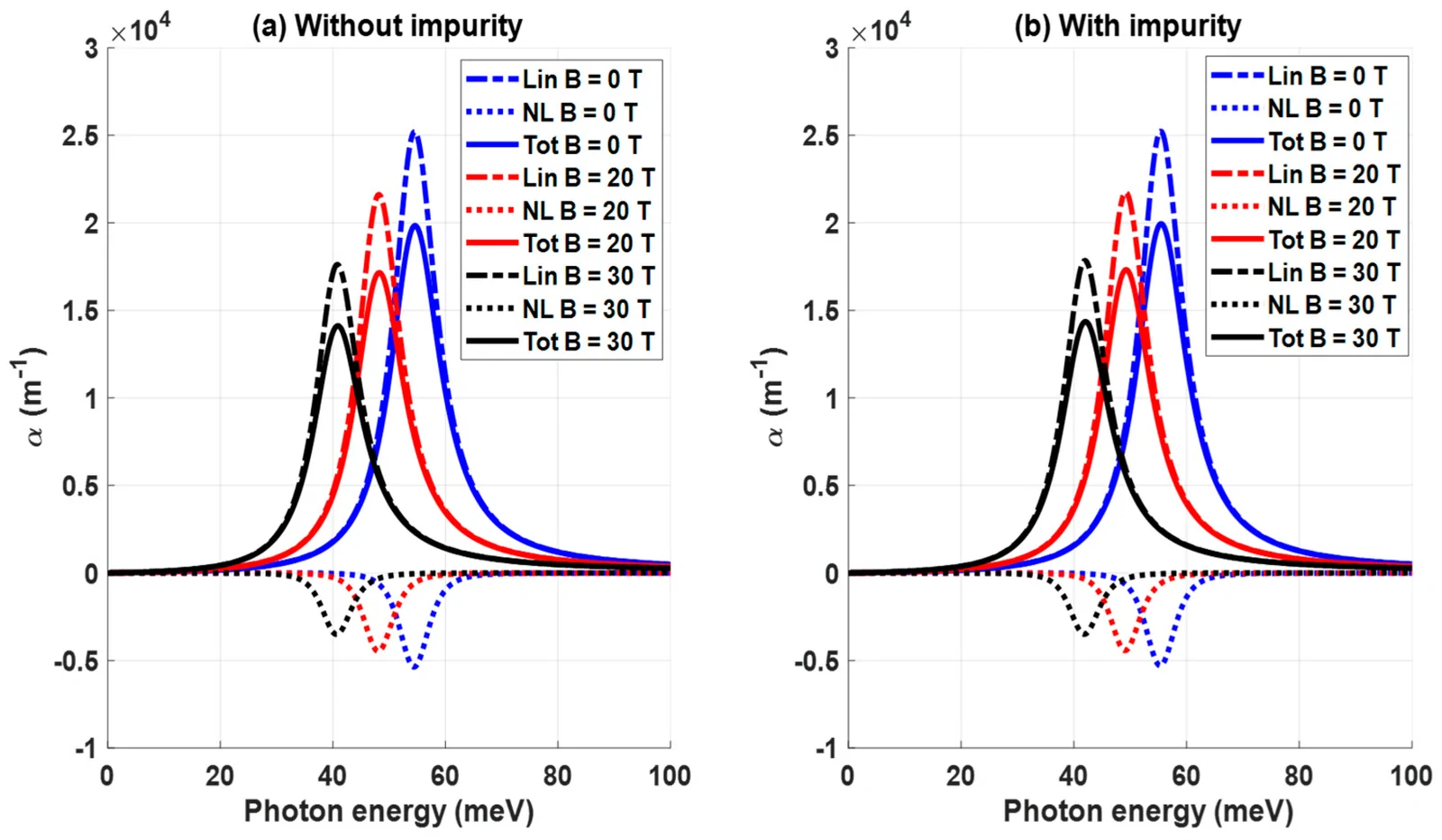
Optical absorption coefficients (linear, nonlinear, and total) as a function of photon energy for B=0, 20, and 30 T (**a**) without the impurity, and (**b**) with the impurity for $R_{1}=5$, $R_{2}=10$, and $R_{3}=15$ nm.

**Figure 9:**
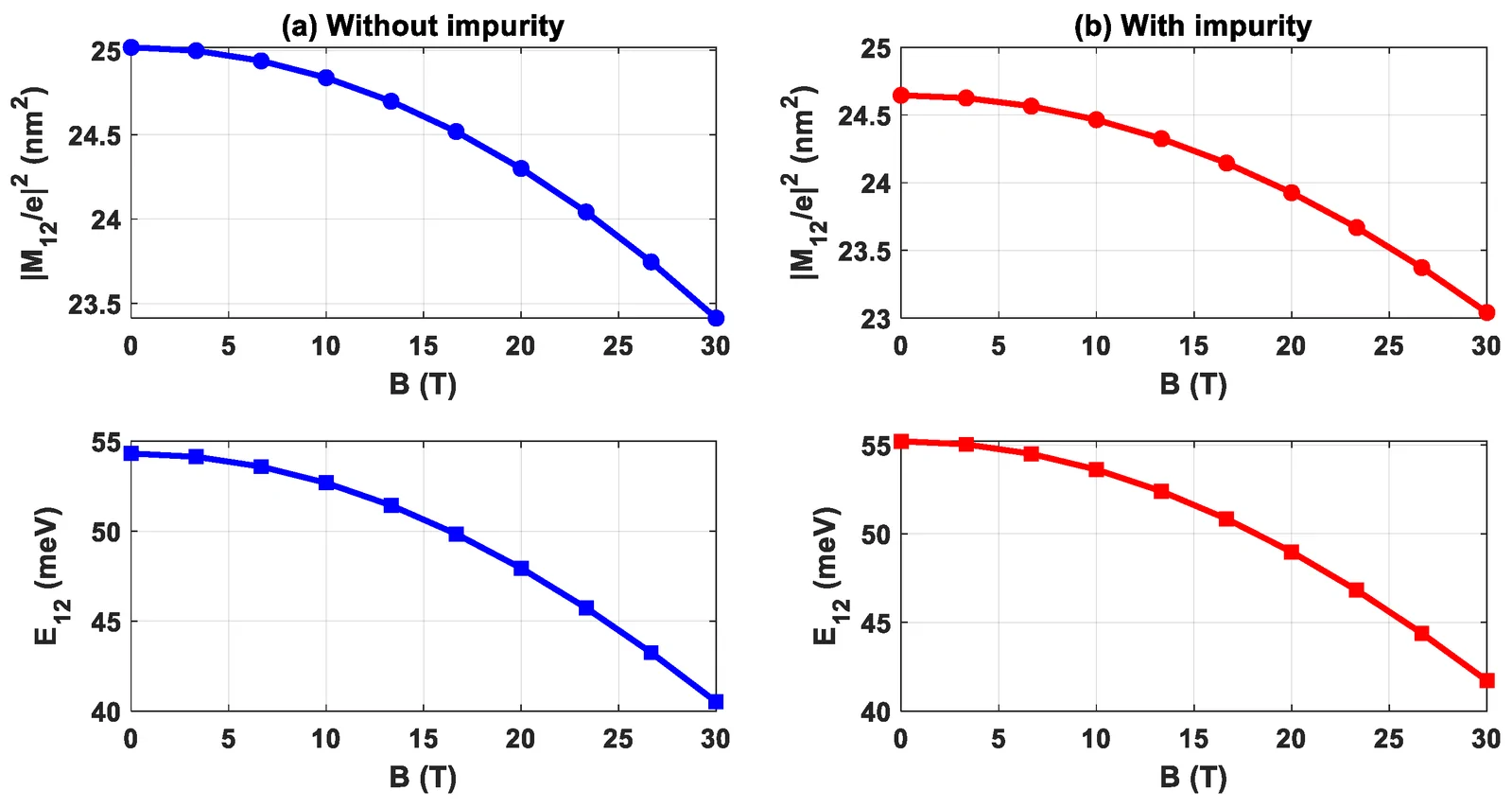
Variation of ${\left|M_{12}\right|}^{2}$ and $E_{12}=E_{2}-E_{1}$ as a function of the magnetic field (**a**) without the impurity, and (**b**) with the impurity for $R_{1}=5$, $R_{2}=10$, and $R_{3}=15$ nm.

Figure 8a,b show the linear, nonlinear, and total optical absorption as a function of the incident photon energy, both with and without the presence of the hydrogenic impurity for B=0, 20, and 30 T. These figures show that both the amplitude and peak positions of these coefficients drop with increasing B. This declining pattern is associated with decreases in the transition energy and the dipole matrix elements, as shown in Figure 9a,b. The redshift observed across all absorption coefficients is a consequence of the reduction in energy separation. The main difference between increasing the internal radius $R_{1}$ and the magnetic field B is that the latter introduces an additional parabolic confinement. The ground-state wavefunctions are greatly localized, and the addition of the magnetic field reinforces their confinement so that their corresponding energy $E_{1}$ displays a small change. However, the wavefunction of the excited state is strongly affected by the magnetic field, such that the two energy levels $E_{1}$ and $E_{2}$ become closer to each other as B increases. Subsequently, the transition energy $E_{12}$ is reduced, which leads to the redshift of the different optical coefficients shown in Figure 8.

**Figure 10:**
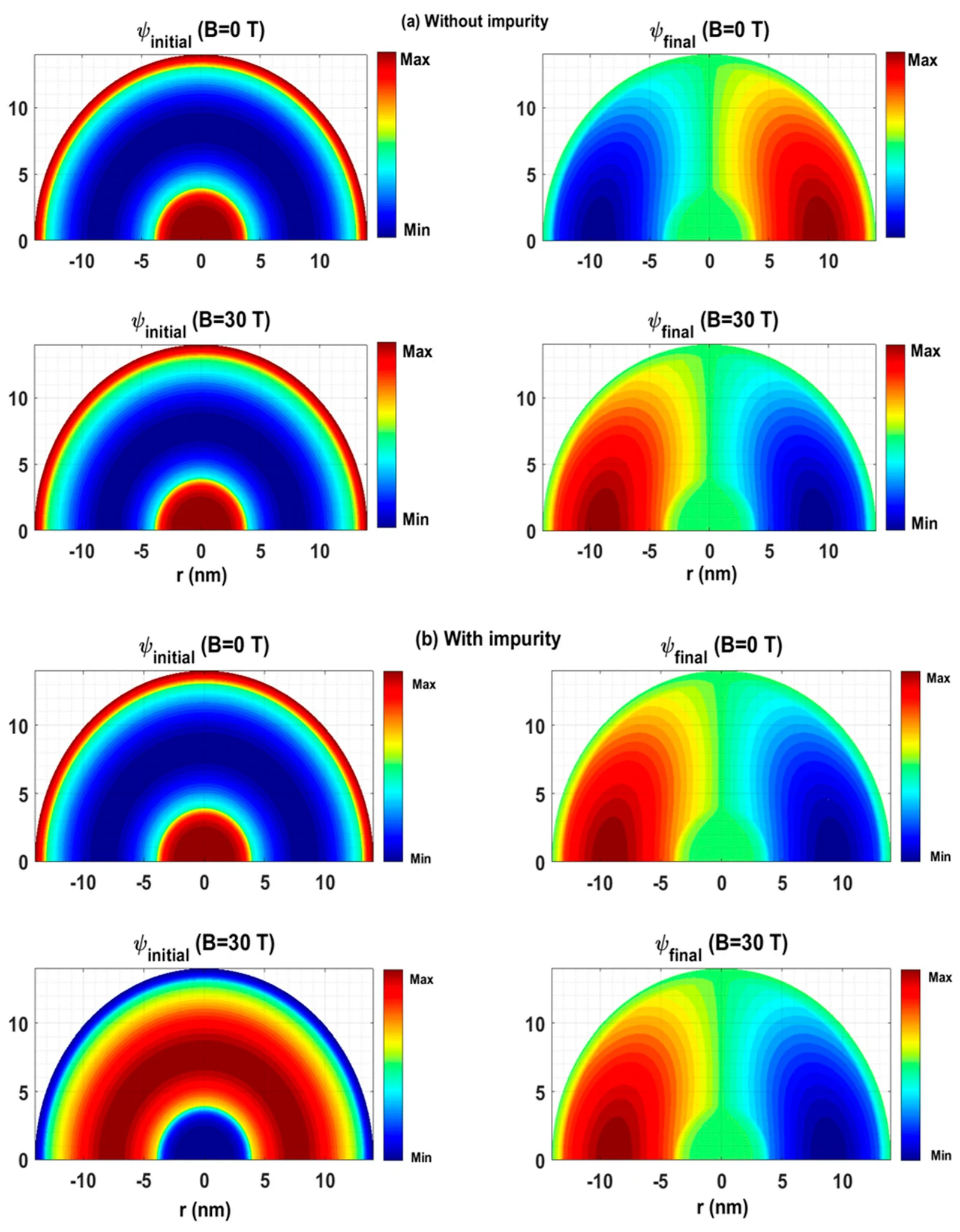
Contour map plots of the initial and final wavefunctions for two magnetic fields (**a**) without the impurity and (**b**) with impurity for $R_{1}=5$, $R_{2}=10$, and $R_{3}=15$ nm.

Figure 10a,b show the wavefunctions for two magnetic field intensities (B=0 and 30 T) with and without the hydrogenic impurity. In the absence of the magnetic field, the ground-state wavefunction exhibits a symmetric spread, primarily due to the spherical character of the confining potential, whereas the excited-state wavefunction exhibits its typical asymmetrical shape of two lobes. As the magnetic field increases, the spatial spread of these wavefunctions changes noticeably due to the additional parabolic confinement imposed by the magnetic term in the Schrödinger equation. This redistribution of electron density is more pronounced in the excited wavefunction than in the ground state. This strong magnetic-field effect on the excited state wavefunction produces a substantial modification of the transition properties, particularly in the dipole matrix element and the optical absorption coefficients. In the presence of an impurity (Figure 10b), the wavefunctions are modified by both the electrostatic attraction and the additional parabolic confinement produced by the magnetic field. The combined presence of these two factors introduces a stronger redistribution of the electronic density.

**Figure 11:**
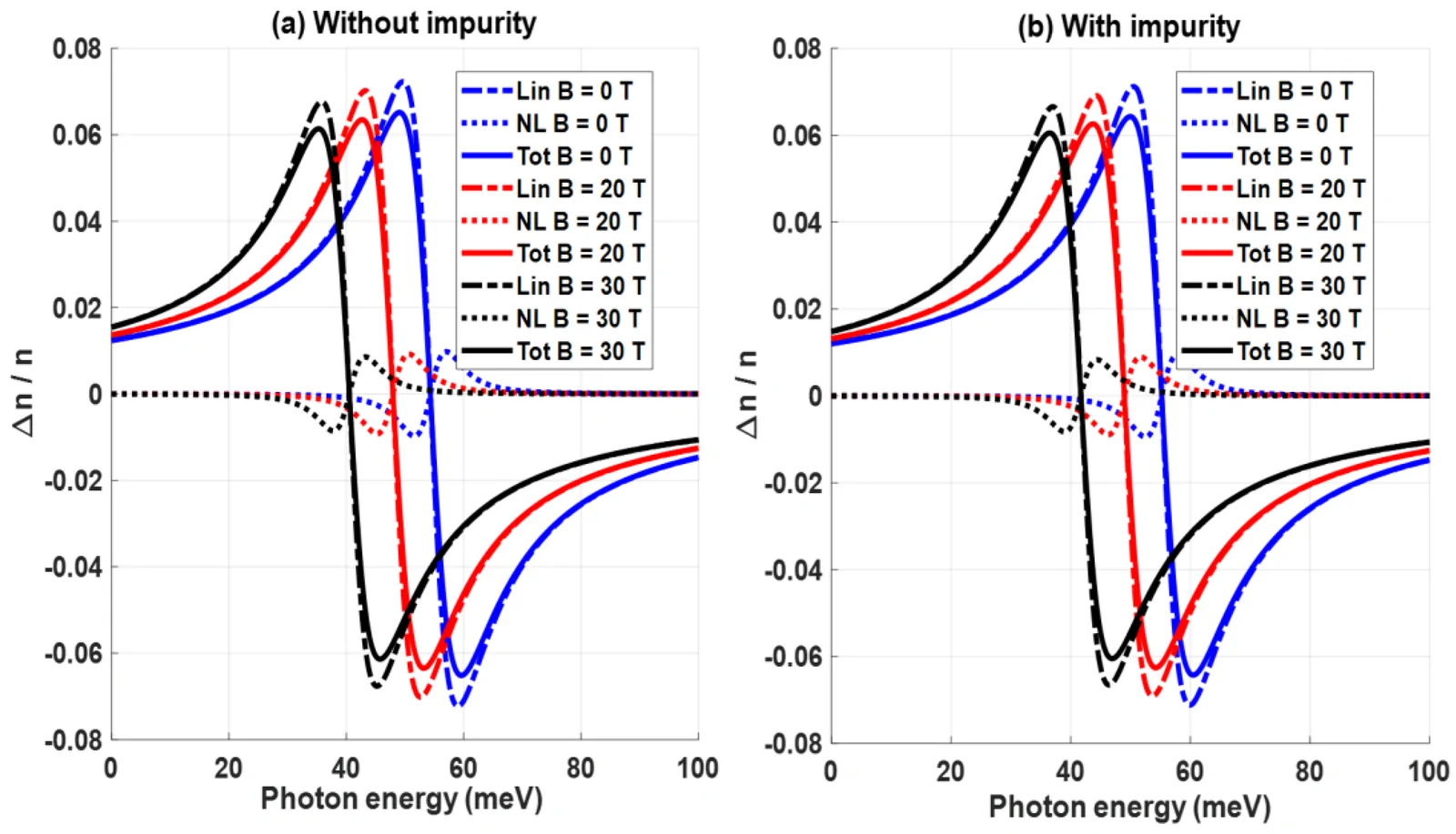
Refractive index changes (linear, nonlinear, and total) as a function of photon energy for B=0, 20, and 30 T (**a**) without the impurity, and (**b**) with the impurity for $R_{1}=5$, $R_{2}=10$, and $R_{3}=15$ nm.

Figure 11a,b plot the three contributions (linear, nonlinear, and total) of the refractive index changes as a function of the incident photon energy for B=0, 20, and 30 T, with and without the presence of the hydrogenic impurity. Increasing the magnetic field shifts all contributions toward lower photon energies, resulting in a redshift. This trend is primarily driven by the decrease in energy transition between the two states. The magnetic field modifies the spatial distribution of the involved wavefunctions, lowering the dipole matrix element and thereby affecting the amplitudes of different refractive-index changes. Note that the nonlinear contribution displays an opposite sign to the linear one, which results in a reduction in the total term. Comparing the two figures, we note that the presence of the impurity introduces a small modification in the magnitudes and positions due to the Coulomb attraction between the impurity and free electrons. This later localizes the carriers around the QD center; however, the redshift is sizeable in both cases.

## 4. Conclusions

In summary, we have examined the optical and electronic properties of a spherical AlSb/InAs/AlSb QD in the presence of an external magnetic field with a hydrogenic impurity. Our results clearly show that the internal radius, *R*_1_, strongly influences the energy spectrum and optical responses, including absorption coefficients and changes in the refractive index. Furthermore, our findings demonstrate that the presence of the hydrogenic impurity lowers all energy levels due to the attractive Coulomb force exerted on the electrons throughout the system. The optical absorption coefficient and refractive index changes were found to be highly sensitive to the internal core dimension and magnetic field intensity. Specifically, increasing $R_{1}$ leads to a redshift due to changes in the all-optical coefficients and the refractive index, primarily caused by a reduction in the energy separation between the ground and excited states and an increase in the dipole matrix element. Analogously, the increase in magnetic field intensity from 0 to 30 T shifts all optical spectra to lower energies, exhibiting a redshift. This trend is due to the additional parabolic confinement produced by the magnetic field. The magnetic effect is more pronounced in the excited-state wavefunction and energy levels. This pattern appears because the first excited-state wavefunction is widely extended in the radial direction and has a greater mean-square radius than the localized ground-state wavefunction. Accordingly, the diamagnetic contribution associated with the magnetic vector potential, as well as the orbital magnetic interaction, produces a larger modification of the excited energy level and spatial distribution. Additionally, our results demonstrate that the nonlinear contributions in both optical absorption and refractive index changes compensate for the linear ones. Consequently, all the total optical responses result from the mutual competition between the linear and nonlinear parts. Overall, the findings presented in this work demonstrate that the optical characteristics of AlSb/InAs/AlSb spherical QDs can be effectively adjusted by monitoring the core/shell dimensions, the presence of a hydrogenic donor impurity, and the application of an external magnetic field. Understanding the effects of these parameters can enable the guided experimental design of QDs with magnetically tunable optoelectronic devices, infrared photodetectors, and nonlinear optical applications.

## Data Availability

The original contributions presented in this study are included in the article. Further inquiries can be directed to the corresponding author.
